# Organizational Factors and Their Impact on Mental Health in Public Safety Organizations

**DOI:** 10.3390/ijerph192113993

**Published:** 2022-10-27

**Authors:** Megan Edgelow, Emma Scholefield, Matthew McPherson, Kathleen Legassick, Jessica Novecosky

**Affiliations:** School of Rehabilitation Therapy, Queen’s University, Kingston, ON K7L 3N6, Canada

**Keywords:** public safety, first responders, occupational health, occupational stress, organizational factors

## Abstract

Public safety personnel (PSP), including correctional officers, firefighters, paramedics, and police officers, have higher rates of mental health conditions than other types of workers. This scoping review maps the impact of organizational factors on PSP mental health, reviewing applicable English language primary studies from 2000–2021. JBI methodology for scoping reviews was followed. After screening, 97 primary studies remained for analysis. Police officers (*n* = 48) were the most frequent population studied. Correctional officers (*n* = 27) and paramedics (*n* = 27) were the second most frequently identified population, followed by career firefighters (*n* = 20). Lack of supervisor support was the most frequently cited negative organizational factor (*n* = 23), followed by negative workplace culture (*n* = 21), and lack of co-worker support (*n* = 14). Co-worker support (*n* = 10) was the most frequently identified positive organizational factor, followed by supervisor support (*n* = 8) and positive workplace culture (*n* = 5). This scoping review is the first to map organizational factors and their impact on PSP mental health across public safety organizations. The results of this review can inform discussions related to organizational factors, and their relationship to operational and personal factors, to assist in considering which factors are the most impactful on mental health, and which are most amenable to change.

## 1. Introduction

Public safety personnel (PSP) work to maintain the safety of civilians and help communities in need [1]. When the terms PSP or first responder are used, the public often think of highly visible occupations, such as correctional officers, firefighters, paramedics, or police officers. While these professions are key stakeholders in the PSP population, it is important to recognize the many other PSP roles including border services officers, rescue personnel, operational intelligence personnel, and communications operators/dispatchers [2,3]. All PSPs take on job demands related to protecting the public, whether it is on the front lines or behind the scenes. Throughout this review, the variety of job demands that PSP face will be referred to as public safety work.

The duties, tasks, and roles associated with public safety work, also thought of as the content of the work, has the potential to expose PSP to psychological trauma [4]. The literature suggests that there is a relationship between PSP work and mental health conditions. Diagnoses including posttraumatic stress disorder (PTSD), depression, anxiety disorders, and substance use disorder, along with suicidal ideation, stress, and burnout, are commonly seen in this population, often at higher rates than the general public [1,4,5].

### 1.1. Impacts of Operational, Personal, and Organizational Factors on PSP Mental Health

There are common operational, personal, and organizational stressors and facilitators that interact with one another to both stress and facilitate the mental health of PSP.

#### 1.1.1. Operational Factors

Operational factors relate to the unique job demands and specific pressures that PSPs face when they fulfill their job [6]. Depending on the situation and the type of PSP worker, traumatic exposures and stressors may present differently. For example, firefighters and paramedics may respond to traumatic events that involve serious injuries to children [7] while others, like policing, may deal with negative public perceptions as an added layer of stress on top of a demanding job [8]. Other operational factors include the volume of work, or workload, and threats to safety and risk of injury or death [6,9]. Additionally, the experience of the COVID-19 pandemic has shown the potential negative mental health impact of increased operational risk when performing public safety duties [10].

#### 1.1.2. Personal Factors

Personal factors are specific to the individual PSP and depending on the person, these factors can act as facilitators or stressors to the complexities that are associated with the specific job. A few important personal factors that the literature shows can add stress to PSP work include having a lack of social support outside of the workplace, or having experiences of childhood trauma [11,12]. In contrast, having a dedicated support system at home and in the community has been shown to act as a personal facilitator for PSP [12].

#### 1.1.3. Organizational Factors

Organizational factors include anything within the employment context that impacts the mental health of PSP during their shifts. These organizational factors may either create added stress or facilitate positive outcomes for the PSP working there [13]. The literature points to some common organizational stressors found among PSP organizations, such as low job autonomy, lack of support from colleagues and supervisors, and shiftwork schedules [6,7,11]. Facilitators can include an increase in job resources and support from supervisors and co-workers, which may lead to higher job satisfaction [6,7].

### 1.2. Need for Further Research

Investigating organizational factors related to mental health is relevant to how well PSP organizations run. If PSP mental health is facilitated, organizational productivity and efficiency levels may improve as well; furthermore, it may create fewer compensation claims or on-the-job accidents [6]. Additionally, it is important to consider operational and personal factors along with the organizational factors. For example, PSPs may be faced with stigma on an operational, personal, and/or organizational level, which may reduce their willingness to seek out mental health services in these organizations and acting on only one type of factor may not fully address needed change [12].

Some reviews have examined operational, personal, and/or organizational factors within PSP organizations, but as separate factors, or within single PSP professions (e.g., [6,11]). However, to date there have not been any reviews completed outlining the most relevant organizational factors, and related operational and personal factors, across public safety professions as a whole. The goal of this review was to map the extent of organizational factors and their mental health impacts in public safety organizations, and to compare this with personal and operational factors.

### 1.3. Review Question

What organizational factors exist in public safety organizations, what are their mental health impacts for public safety personnel, and how does the frequency of organizational factors compare with personal and operational factors?

## 2. Materials and Methods

This review was conducted using the Joanna Briggs Institute (JBI) methodology for scoping reviews [14]. We used the Preferred Reporting Items for Systematic Reviews and Meta-Analyses extension for Scoping Reviews (PRISMA-ScR) [15].

### 2.1. Inclusion and Exclusion Criteria

This review considered studies that included public safety personnel participants, including but not limited to police officers, correctional officers, emergency dispatchers, firefighters, and paramedics. The review excluded studies that did not specifically report on public safety personnel. Studies that reported on organizational factors were included, and those that did not were excluded.

This review considered studies that were conducted in the context of public safety organizations, including but not limited to communications and emergency dispatch departments, correctional facilities, fire departments, paramedic departments, police departments, and search and rescue organizations. Studies that did not take place in a public safety setting were excluded.

All genders of participants and types of public safety organizations were included; only Anglocentric nations were included (Australia, Canada, New Zealand, United Kingdom, and United States) to ensure commonalities in the context of the public safety work.

### 2.2. Types of Sources

Primary research that reported on the impact of organizational factors on mental health in public safety organizations was considered. Where operational and personal factors were present within these studies, they were also considered. Primary research was the focus of this scoping review to specifically understand the factors and their impact.

Both experimental and quasi-experimental study designs, descriptive observational study designs, analytical observational studies, and qualitative studies were considered for inclusion. As detailed in Section 2.1, only studies from Anglocentric nations were considered for this review due to similarities in public safety working conditions, and so only articles published in English were included. Papers were restricted to the current century, including the years 2000–2021, to ensure that the studies most relevant to current public safety practices and work contexts were found.

### 2.3. Search Strategy

The search strategy located published primary research studies. An initial limited search of PsycInfo and MEDLINE, on the Ovid platform, was undertaken to identify initial articles on the topic. The text words in the titles and abstracts of these relevant articles, and the index terms used to describe the articles, were then used to develop a full search strategy for MEDLINE. This search strategy, including all identified keywords and index terms, was adapted for each included information source or database (see Appendix A for a sample search strategy).

### 2.4. Information Sources

The databases searched were Embase, PsycInfo, and MEDLINE, on the Ovid platform, as well as Web of Science on the Clarivate platform, and CINAHL on the Ebsco platform, to ensure a broad search for relevant studies.

### 2.5. Study Selection

**Figure 1:**
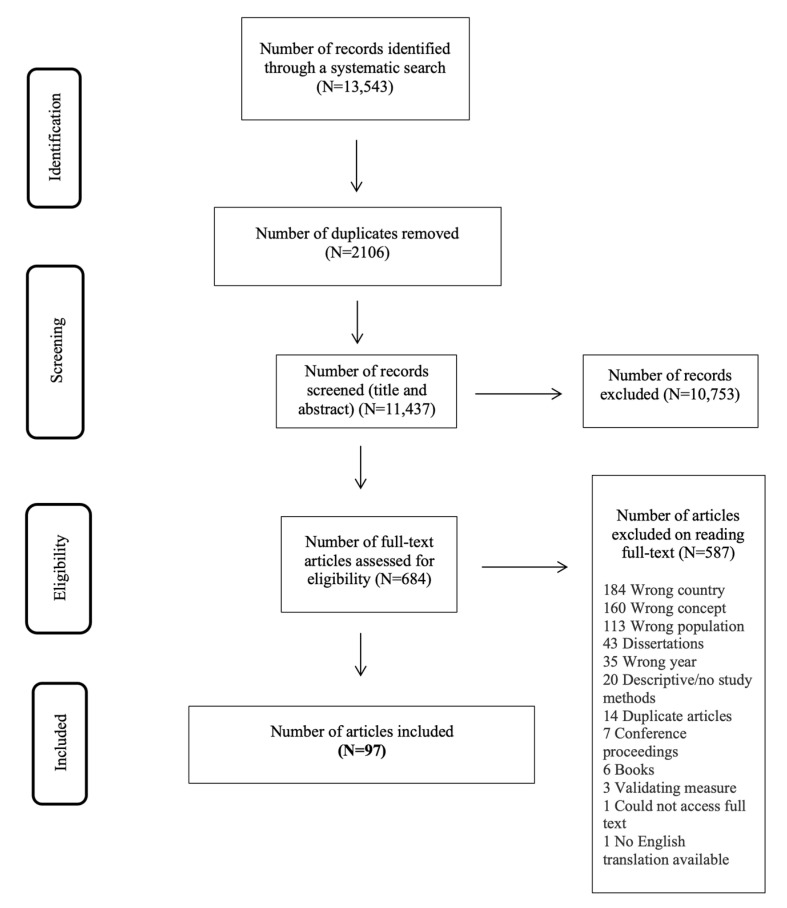
PRISMA flow diagram of search and study selection process [16].

Following the search, all identified citations were collated and uploaded into the Covidence platform, which removed duplicates. Titles and abstracts were screened by four independent reviewers and assessed against the inclusion criteria for the review, using Covidence. Potentially relevant studies were retrieved in full, and their citation details imported into Covidence for further review. The full text of selected citations was assessed in detail against the inclusion criteria by the same four independent reviewers. The reasons for exclusion of full text studies not meeting the inclusion criteria were recorded and reported in Figure 1. Disagreements that arose between the reviewers at any stage of the study selection process were resolved through discussion, or with a fifth reviewer. The results of the search are presented in a Preferred Reporting Items for Systematic Reviews and Meta-analyses (PRISMA) flow diagram (Figure 1) [16].

### 2.6. Data Extraction

Data was extracted from articles included in the scoping review by four independent reviewers using a data extraction tool developed by the reviewers. The data extraction tool was in table format and included columns for the population, concept including personal, operation and organizational factors, context and additional details, study methods and key findings relevant to the review objective. Disagreements that arose between the reviewers were resolved through discussion, or with the fifth reviewer.

## 3. Results

### 3.1. Study Inclusion

After the initial search, 13,543 articles were found, with deduplication, 11,437 remained, with 684 of these meeting screening criteria. After full-text access and review, 587 articles that did not meet inclusion criteria were eliminated, leaving 97 articles for inclusion in this review (Figure 1).

### 3.2. Characteristics of Included Studies

#### 3.2.1. Study Design Characteristics

Table 1 outlines the study characteristics and factors of the 97 papers that were identified through the search process. 60 percent (*n* = 58) of the papers were published in the last five years. Most included studies were conducted in North America: United States (*n* = 43) and Canada (*n* = 19). Other included studies were conducted in the United Kingdom (*n* = 19), Australia (*n* = 16), and New Zealand (*n* = 2). One study was conducted within Australia and the UK, another within the United States, Canada, and Europe, and another included Canada and the United States. Two-thirds of the studies utilized cross-sectional research designs (*n* = 62). Qualitative (*n* = 15) and cohort studies (*n* = 5) were the next most frequent study designs. Other study designs included: longitudinal (*n* = 4), mixed methods (*n* = 4), quasi-experimental (*n* = 3), case–control (*n* = 2), and randomized control trial (*n* = 1) designs. Finally, one study measured the psychometric properties of a measurement tool.

#### 3.2.2. PSP Population Characteristics

Police officers (*n* = 48), including Royal Canadian Mounted Police (RCMP) officers (*n* = 3), online police investigators (*n* = 2), a child abuse investigator (*n* = 1), municipal/provincial police (*n* = 1), federal police (*n* = 1), and transit police (*n* = 1) were the most frequently identified population studied. Correctional officers (*n* = 27) were the second most frequently identified population, including correctional supervisors (*n* = 2) and a parole or probation officer (*n* = 1), tied with paramedics (*n* = 27), which included emergency medical technicians (EMTs; *n* = 10) and emergency medical services (EMS; *n* = 1). Career firefighters (*n* = 20), dispatchers (*n* = 5), volunteer firefighters (*n* = 4), communication officers (*n* = 3), coast guard (*n* = 1), Canadian border services (*n* = 1), State emergency service employees (*n* = 2), and immigration detention staff (*n* = 1) were less frequently studied. Eleven studies included more than one PSP group within the study population (corrections *n* = 3, dispatchers *n* = 5, fire (career) *n* = 8, fire (volunteer) *n* = 4, paramedics *n* = 8, police *n* = 9, other police *n* = 6). Finally, population groups ranged from five to 21,160 participants. Most studies included between 100 to 249 participants (*n* = 21), 250 to 499 (*n* = 20), and 1000 to 2499 (*n* = 20).

### 3.3. Operational, Organizational and Personal Factors

While the literature search focused on organizational factors, operational and personal factors were frequently reported by the same papers and so were also captured during data extraction. Table 1 also outlines the positive and negative operational, personal, and organizational factors found in the included studies. Below are the most common factors extracted from the 97 articles included in this scoping review. Numbers in [brackets] refer to the article identification numbers of Table 1. Please note that some articles reported on organizational factors, but the factors were deemed neither positive nor negative (e.g., neutral factors or factors without evidence of impact). These articles are included in the review as they met inclusion criteria but may not have data noted in Table 1 related to positive or negative organizational factors.

#### 3.3.1. Positive Factors

##### Positive Operational Factors

Operational factors are considered to be the unavoidable aspects of public safety work, sometimes referred to as the content of the work. Two articles identified a positive relationship between work role and mental health outcomes [93,100]. For example, police officers working in “Operational Support” roles demonstrated lower odds of developing PTSD compared to investigations officers [100]. In another study, correctional workers in Institutional Governance (e.g., superintendents, and correctional managers) had higher mean PTSD scores than those working in Institutional Wellness (e.g., nurses, social workers, psychologists, etc.) [93]. Furthermore, participants working in institutional correctional services demonstrated higher problematic alcohol use scores than participants working in Institutional Wellness, Institutional Administration (e.g., administrative assistants), and Community Supervision Officers (e.g., parole and probation officers) [93]. Department setting (*n* = 1) was another positive operational factor; working in suburban, urban, and mixed departments was associated with a lower risk of depression, PTSD, and suicidality scales compared to rural departments [56]. Finally, in one study, tenure and rank showed a positive relationship with PSP mental health [56,82].

##### Positive Personal Factors

Personal factors refer to factors unique to the individual performing the public safety work; these factors exist outside of the work context but may interact with it. Out of the 97 included studies, family support (*n* = 8) [5,18,21,41,45,46,48,77] and job satisfaction or meaning (*n* = 8) [5,19,26,43,77,100,105] appeared to be the most frequent supportive personal factors. For example, one article emphasized the protective role of family relationships in preventing correctional officers from attempting suicide [48]. Regarding job satisfaction and meaning, another article identified police officers as obtaining satisfaction through contributing positively to the lives of civilians in disaster-struck communities [22]. Indigenous correctional officers found meaning in supporting the rehabilitation of Indigenous prisoners [90]. Four articles listed work/life/family balance (*n* = 4) [27,43,46,105] and gender (*n* = 4) [22,71,79,106] as supportive personal factors. In one article, female PSPs reported lower levels of PTSD compared to their male counterparts [22]. Adequate sleep (*n* = 3) [27,42,100], including sleep quality and sleep duration, demonstrated a positive influence on PSP mental health in three studies. Several positive coping skills (*n* = 3) [46,83,85] were noted, including going for long walks, participating in yoga and meditation, accessing psychological supports, doing exercise, abstaining from alcohol, detachment from work issues, problem-solving, pondering, and lack of effective rumination [43,46,83]. Resiliency (*n* = 3) [8,75,108] was associated with lower secondary traumatic stress in PSP populations [75]. Finally, good physical health (*n* = 3) [43,76,85], race (*n* = 3) [71,82,106], and social support (*n* = 3) [18,29,101] were other identified positive factors.

##### Positive Organizational Factors

Organizational factors refer to the context in which public safety work occurs. Co-worker support (*n* = 10) was the most frequently identified organizational factor that facilitated PSP mental health [26,37,39,43,45,46,49,75,77,85]. Supervisor support (*n* = 8) was the second most common positive organizational factor identified [23,39,45,47,49,62,66,85]. Four studies listed autonomy as an organizational factor that promotes PSP mental health [45,83,84], while adequate training [39,43,46] and workplace culture [23,60,62] were included in three studies. Access to mental health specialists [39,48] and good leadership [52,66] were organizational facilitators included in two different studies. For example, correctional workers who accessed ongoing treatment achieved and maintained mental wellness [48]. Recognition of good work (*n* = 2) was another organizational factor that facilitated first responder mental health [19,52]. Finally, role clarity [83,96] and team dynamics [23,37] were included as positive organizational factors in two studies, respectively.

#### 3.3.2. Negative Factors

##### Negative Operational Factors

Exposure to critical incidents [5,19,22,24,35,37,39,40,41,52,53,64,67,71,78,82,89,90,92,103,104] was the most frequently cited negative operational factor, included in 21 studies. High workload [8,19,29,31,32,33,36,45,46,47,57,59,66,70,73,74,80,96,98] was the second most frequently cited negative operational factor (*n* = 20). Thirteen articles identified threats or risk of violence [41,44,45,50,66,69,70,76,77,81,92,94,103,104]. Administrative duties (e.g., paperwork) (*n* = 12) [5,19,30,44,50,53,74,80,82,86,91,108], negative public perception of career (*n* = 12) [5,21,30,37,41,44,69,70,73,82,91,108], workplace stress (*n* = 12) [24,50,69,70,77,78,82,95,96,100,101,108], and risk of injury to PSP (*n* = 12) [5,21,35,40,43,44,50,69,70,77,82,92] were each included as negative operational factors. Nine articles listed experiencing violence [32,41,49,51,66,67,68,82,92] while eight cited work overload [37,43,44,53,72,73,77,96] as negative operational factors. Finally, risk of death [35,44,64,77,94] was a frequent but less commonly identified (*n* = 5) operational factors negatively impacting PSP mental health.

##### Negative Personal Factors

Experiencing mental health conditions was the most frequently cited (*n* = 26) negative personal factor, included in 27 percent of articles [22,24,26,29,30,31,34,35,40,47,50,51,52,53,55,59,67,77,80,85,90,92,98,100,101,107]. PTSD, anxiety, depression, and general mental health difficulties were the most frequently listed diagnoses. Work/life/family conflict was the second most common negative personal factor, identified in 20 percent of articles (*n* = 19) [5,21,22,31,33,37,40,41,47,48,57,63,69,70,77,82,95,96,111]. Two articles described conflicts between work and family as sources of PSP stress [21,111]. Public safety careers also impacted PSPs ability to maintain a social life outside of work [40] due to the “unsociable” working hours and lack of time outside of working hours [5,57]. Another article found an association between work–life conflict and burnout in firefighters [95]. The following three factors were identified in 12 articles each: gender [18,25,36,47,49,51,55,56,79,102,106,111], poor sleep [18,29,34,41,42,43,50,67,69,84,86,98], and job dissatisfaction [28,31,33,50,55,59,73,80,81,89,104]. Fatigue (*n* = 9) [5,8,21,30,34,40,84,86,98], lack of coping skills (*n* = 10) [8,26,33,39,41,43,50,67,85,92], health conditions (physical) (*n* = 8) [26,31,35,47,52,77,85,107], substance misuse (*n* = 8) [18,41,43,47,62,65,76,105] and burnout (*n* = 7) [26,29,32,36,67,80,97] were also identified, but were less common overall.

##### Negative Organizational Factors

Lack of supervisor support was the most frequently cited negative organizational factors (*n* = 23) [25,30,31,32,33,43,44,47,50,59,70,71,73,77,80,81,82,88,91,92,98,101,111]. Over one-fifth of articles (*n* = 21) identified workplace culture [5,23,28,31,33,35,37,38,41,49,55,71,74,80,81,82,91,92,105,106,112] as a negative organizational factor contributing to poor mental health outcomes in PSP. Fifteen percent of articles (*n* = 14) cited lack of co-worker support [22,25,47,50,59,65,71,75,82,86,88,90,92,111] and limited resources to perform the work [5,23,46,53,71,73,76,77,82,83,88,91,95,96]. Furthermore, interpersonal conflict with a colleague [5,25,28,47,65,76,77,80,82,83,86,91,108] and stigma/barriers to seeking help [5,32,35,37,38,39,43,48,49,51,67,77,89] cited in 13 studies each. Finally, leadership issues [5,30,31,37,43,57,73,77,81,82,86] overtime hours [5,8,20,29,37,43,51,57,67,69,82,84,110], and understaffing [5,32,45,74,77,80,81,82,90,91,96,108] were identified as negative organizational factors in 12 studies each. Eleven articles cited various models of shift work [19,24,29,40,53,77,78,82,84,85,98] as negatively impacting PSP mental health outcomes.

### 3.4. Factor Frequencies

After completing the extraction of the 97 articles and determining meaningful characteristics of the data, this scoping review gathered a total of 607 positive and negative factors within the operational, personal, and organizational factors found in PSP organizations (Table 2). A total of 126 operational factors (negative factors = 119; positive factors = 7), 273 personal factors (negative factors = 206; positive factors = 67), and 208 organizational factors (negative factors = 145; positive factors = 63) were found. Negative factors were discussed a total of 470 times and positive factors were discussed a total of 137 times.

## 4. Discussion

In the completion of this review, the frequency of factors, trends across countries, specificity of factors to PSP groups, and the amenability of organizational factors to change were most notable and will be discussed below.

### 4.1. Factor Frequencies

Table 2 shows the frequencies of the negative and positive operational, personal, and organizational factors discussed in the evidence outlined in this scoping review. Based on these frequencies, negative factors (*n* = 470) were much more prevalent than positive factors (*n* = 137). This could be a result of how the studies were constructed; researchers may have found that discovering the barriers impeding PSP mental health is a more pressing issue to discuss as opposed to the positive aspects of public safety work. It is also possible that negative factors and stressors are more commonly seen in PSP organizations than positive factors and facilitators to mental health.

This review focused on discovering what organizational factors exist in public safety organizations and how the frequency of organizational factors compare with personal and operational factors. It is interesting to note that personal factors, both negative and positive, were more commonly seen (273 times) than organizational factors (208 times). Personal factors accounted for a total of 45 percent of factors found, which was often due to studies reporting mental health conditions (*n* = 26), issues of work/life/family conflict (*n* = 19), physical health and sleep concerns (*n* = 33), as well as demographic factors (*n* = 12). Because the focus of this review is on organizational factors, the impact of personal factors will not be discussed in further detail but the factors themselves can be found within Table 1 for further information.

Comparing all the factors that were discussed, organizational factors accounted for 34 percent with operational factors at 21 percent, respectively. This data shows that the context and content of public safety work may be responsible for over one half of the factors influencing PSP mental health, and therefore public safety organizations have impactful opportunities to change these factors.

### 4.2. Trends across Countries

Another trend to consider from this scoping review is the frequency of PSP research in each country. In our review we found that North America (United States and Canada) was the primary region that PSP research was being recognized and conducted. Policing, firefighting, and corrections were being studied primarily in the United States and Canada, whereas other PSP professions such as paramedics and EMTs were being studied primarily in Australia. There has been limited research in communications officers and dispatch throughout all the countries investigated in this review. Canada, the United States and the United Kingdom were the most frequently cited countries in this review, therefore, the recommendations mentioned below may be most applicable to these countries.

In this review, negative organizational factors were most commonly studied in the United States. These studies frequently found a lack of supervisor support, negative workplace culture, limited access to resources to do the work, lack of co-worker support and various shiftwork models as negative organizational factors in PSP organizations. Stigma was seen most in Canadian studies and leadership issues were most prominent in the studies from the United Kingdom. This may indicate more attention to these issues in these countries, rather than an increased presence of the issues in these places.

### 4.3. Common Operational and Organizational Stressors Impacting PSP

Given that the content and context of the work performed within public safety organizations is a potential focus for employers related to mitigating risks to their employees, the most frequent operational and organizational factors will be discussed by career type, in order of their frequency of appearance in this review.

#### 4.3.1. Police Officers

Negative public perception of career [5,20,30,37,44,69,70,82] and risk of injury [3,5,20,35,43,50,69,70,82] were the primary operational factors negatively impacting police officer mental health outcomes. Loughran [113] explained that a string of recent high-profile cases of police violence may have eroded public perception of police legitimacy. Negative public image is an organizational stressor police officers frequently face [110]. Increased public scrutiny and animosity towards police negatively impact individual encounters between officers and citizens. Poor interactions with the public can worsen police officers’ mental health outcomes [37].

In terms of organizational factors, workplace culture was the most frequently encountered negative factor. Demou et al. [37] identified that police officers in particular “are afraid of being identified as individuals who have been compromised by stress” (p. 703). Coworker support [26,37,39,43,45,46,49,75,77,85] and supervisor support [23,39,45,47,49,62,67,85] were the most frequently cited positive factors. These factors were included more often than any other factor. Dollard et al. [39] explained that in workplaces with a high psychosocial safety climate, police officers know they will be supported in the face of unexpected demands (e.g., attending a fatal shooting or vehicle accident). This support can improve coping ability, which serves a protective role against negative mental health outcomes [39]. The findings of this review were congruent with a 2017 literature review of police stressors and their impact on health [8], which showed that both mental and physical health impacts were associated with organizational stressors in police organizations.

#### 4.3.2. Correctional Officers

Operational factors were not as commonly identified in the articles found for correctional officers. Articles listed psychological demands [25,29] and high workload as negative operational factors. Perceived threat to safety [72] and role [93] were the only operational factors identified which positively influenced mental health outcomes for this population.

Workplace culture was the most frequently identified negative organizational factor for correctional officers [5,38,41,80,81,92,105,112]. Dugan et al. [41] noted that correctional officers operate in a masculine culture, where personnel are expected to display strength and control and suppress emotions. Several participants in the Norman & Ricciardelli [80] study identified management as the source of toxic workplace culture. They elaborated that leadership engaged in deception and deceit with their employees, leading many to leave meetings with headaches and in tears [80]. Stigma and barriers to seeking help [5,32,38,48,67,89,90] and understaffing [5,32,45,80,81,90] were the next organizational factors negatively impacting mental health outcomes. Clements & Kinman [32] explained that “the reporting of mental health challenges is stigmatized in ‘macho’ types of work” (p. 444). This leads correctional officers to under-report stress [32]. Interpersonal conflict with a colleague was also a source of stress [5,25,65,80]. Support from coworkers [26,45] and supervisors [45,67] were the primary positive organizational factors for COs. Buden et al. [26] suggested that workplace social support (from coworkers and supervisors) promotes health behaviour change (e.g., increased sleep duration). Autonomy [45] and access to mental health specialists [48] were also positive organizational factors. A systematic review of correctional officer job stress and burnout [114] also showed the strong impact of workplace culture on mental health for correctional officers.

#### 4.3.3. Paramedics and EMTs

High workload [36,73,74,85,96,98] and incidents involving children [22,77,85,86] were the most frequently included negative operational factors. Sofianopoulos et al. [98] identified that paramedics are, “constantly and increasingly faced with difficult clinical cases and workload that are taxing physically, mentally, and emotionally” (p. 2). Mahony [74] described how a reduction in the number of on-road paramedic crews, combined with increased emergency call volumes, has led to a process of work intensification in the UK context. This results in paramedics having little to no down-time between calls, where they would typically decompress back at the station [74]. Department setting and rank [56] were the only operational factors included which positively influenced paramedic mental health.

Limited resources to perform the work [73,77,88,91,96] was the most common organizational factor negatively impacting paramedic and EMT mental health. Similar to firefighters, Mahony [73] identified that paramedics were, “constantly pushed to achieve more with less resources” (p. 141). Lack of control over resource provision also negatively influences PSP mental health [73]. Navarro Moya et al. [77] further explained that economic crisis has resulted in significant cuts to resources. Workers may dissociate themselves from their role when an organization does not provide the necessary economic or emotional resources [77]. Lack of breaks while working [73,74] and other models of shift work [40,98] were additional organizational stressors, included in two articles each. Regarding positive organizational factors, coworker support [77,85] appeared most frequently. Supervisor support [85], role clarity [96], a 12-h shift model and a 14-h shift model [56] were also included. Organizational and operational factors faced by paramedics and EMTs did not differ significantly. A 2019 systematic review [7] focused on ambulance personnel showed how operational and organizational factors can interact to amplify job stress, and the value of supervisor support and positive leadership, supporting the current findings of this review.

#### 4.3.4. Firefighters (Career and Volunteer)

Regarding negative operational factors, exposure to critical incidents [5,18,63,90] was the most frequently cited factor. Langtry et al. [64] noted that firefighters described feeling “locked in” a cycle of perpetual traumatic exposure. Firefighters are often expected to return to an “operationally ready” state after returning from an emergency deployment [64]. Armstrong [19] suggested that providing positive reinforcement after critical incidents might buffer this stressor. Department setting and rank [56] were the only positive operational facilitators identified.

Limited resources to perform the work [5,76,82,91,95,96] was the most frequently identified negative organizational factor. Smith et al. [96] identified that line-of-duty operations (such as firefighting) are expected to be performed flawlessly despite limited available resources. These expectations elicit strain on firefighter mental health. Smith et al. [96] further explained that providing adequate resources would alleviate stress and burnout. Interpersonal conflict with colleagues and supervisors [76,82,91] were the next factors negatively impacting firefighter mental health outcomes. In terms of positive organizational factors, safe work practices [97] and various models (e.g., 12, 14, 48 h) of shift work [27,56] were considered. No differences in organizational/operational factors were observed between career and volunteer firefighters. A recent meta-analysis of the impact of organizational support [115] has shown that attending to working conditions can improve employee well-being, supporting the relevance of attending to firefighters’ resources to perform their duties, as well as the interpersonal aspects of their work.

#### 4.3.5. Communications Officers and Dispatchers

In terms of negative operational factors, exposures to critical incidents [5,24], administrative duties, and negative public perception of career [5,91] were the next most frequently associated with communications officers’ and dispatchers’ mental health. There were no positive operational factors identified in the literature for this population, but the literature overall for this PSP group was limited (*n* = 8).

Workplace stress [101], lack of support from supervisors [47,91,101] and coworkers [47,75,90] were the primary negative organizational factors faced by communications officers and dispatchers. Galbraith [47] suggested that call and dispatch departments have different expectations for leadership than operational police officers. This study identified that managerial support was severely lacking, poorly impacting personnel’s mental health outcomes [47]. Birze et al. [24] explained that communications officers, “must regularly balance and control their emotional reactions, both in themselves and others” when taking calls, dispatching, and interacting with co-workers or supervisors (p. 426). Communications officers, therefore, engage in surface acting, which involves, “hiding unsuitable feelings and faking inauthentic-yet organizationally prescribed feelings” [24] (p. 426). However, surface acting with colleagues and supervisors and colleagues was associated with higher reported PTSD symptoms [24]. Stigma and barriers to seeking help [5,90] were also negative organizational factors. Supervisor support [47] was the only positive organizational facilitator included. A recent qualitative study of public safety communications professionals [116] showed that leadership, supervision and workplace culture are key factors in the well-being of this PSP group, further supporting the findings of this review.

Other PSP workers (e.g., border services officers, rescue personnel, operational intelligence personnel) were not found with enough frequency by this review to analyze them in detail by profession, and were under-represented overall within the literature found.

### 4.4. Factors Amenable to Change

Ricciardelli et al. [9] point out that organizational factors (e.g., job context) in comparison to operational factors (job content) were higher sources of stress and seen to be avoidable factors in a PSP context. The operational risks of PSP duties generate stress and anxiety in workers, but, since they are often inherent to the job, they can be unavoidable. The amenability to change of organizational and operational factors were also considered by Carleton et al. [5] in assessing the impact of work stressors on PSP, and are worthy of further consideration here. Of all the factors found in this review, evidence suggests that supervisor support, leadership styles, shift work models, staffing levels, stigma, and workplace culture are just some of the characteristics of PSP organizations that may be amenable to change.

#### 4.4.1. Supervisor Support

Supervisor support appeared frequently within this review [25,30,31,32,33,43,44,47,50,59,70,71,73,77,80,81,82,88,91,92,98,101,111]. Vaughan et al. [117] highlighted how supervisor support can be improved in PSP organizations by establishing closure for workers, empowering immediate supervisors, and changing the organizational culture. These changes may differ in each organization or discipline but overall, supervisors must consider the needs and long-term mental health status of their workers. For example, Stanley et al. [118] concluded that supervisor social support (top-down mental health promotion) could be beneficial to the health and well-being of firefighters. Additionally, immediate supervisors can be empowered to advocate on behalf of their workers for more support services, and they can incorporate increased cultural awareness in recruitment and ongoing training [117].

#### 4.4.2. Leadership Styles

Leadership [5,30,31,37,43,57,73,77,81,82,86] in PSP organizations is presumed to mold the efficacy within the organization, improve the behaviour of the employees, and increase outputs for the agency [119]. With leadership style having such potential impact, leadership is important to address, potentially by implementing a development program for leaders. Leadership development programs should promote practices that focus on support strategies and visions of empowerment to increase engagement from the employees and supportive work resources [120]. However, one of the major barriers to implementing successful leaders and leadership processes includes the cultural, structural, and political aspects of the organization [119]. To implement changes in leadership style that are sustainable and effective, it is important to first address these higher-level underlying barriers that underly the organization.

#### 4.4.3. Shift Work Models

Shift work [19,24,29,40,53,77,78,82,84,85,98] appeared frequently within this review, and can create difficulties for PSP as they may promote lack of sleep, which can further increase the risk for disease and/or workplace injury [8,9]. Changing the way shift work is implemented, such as adapting the schedule to also consider the PSP’s personal and family needs may decrease negative health outcomes [121]. The length of a shift could be changed to provide more desirable outcomes for the PSP and the organization they work for. For example, Amendola et al. [17] found that there was a benefit to implementing 10-h work shifts over 8- or 12-h work shifts. When 10-h work shifts were implemented, PSPs reported a higher quality of life during work and increased sleep overnight. They also found that when completing 10-h shifts, employees completed less overtime, which could potentially be a cost-saving strategy as well. With increased hours of sleep, PSPs may see a reduced risk of mental health challenges and/or workplace injury. Given the variety of work contexts for different PSP groups and the lack of current conclusive evidence to promote a singular shift model, organizations might look to their employees and their unions to understand worker needs and preferences.

#### 4.4.4. Staffing Levels

Staffing levels were identified frequently within this review as an important organizational factor [5,32,45,74,77,80,81,82,90,91,96,108]. Ricciardelli [9] recommended that promoting work–life balance and allowing more time-off must be encouraged as both preventative and reactive measures to reduce work overload and stress among PSP workers. The challenge with this change is the need for more funding to improve staffing shortages. If PSPs are not given the opportunity to take needed breaks, they may be more susceptible to unexpected stress leaves, placing added pressure on the remaining staff [122]. The combination of work overload and limited organizational staffing may contribute to burnout amongst all staff members and increased organizational costs overall, making these factors a priority for change.

#### 4.4.5. Stigma and Workplace Culture

The stigma surrounding accessing mental health resources or treatment is another barrier in PSP organizations that can negatively impact the mental health of PSPs [5,32,35,37,38,39,43,48,49,51,67,77,89]. Vaughan et al. [117] found that organizations could see positive results with workplace culture that emphasizes the mental health of their staff. Given that lack of supervisor support was the most frequent negative organizational factor, and co-worker support was the most frequent positive organizational factor, efforts to improve workplace culture, reduce stigma, and provide organizational supports could all contribute to an improvement of this area [5].

### 4.5. Limitations

This study used a scoping methodology, and thus, the rigour of the included sources was not evaluated. Additionally, it is possible that searching a separate set of databases, or a different date range, may have identified additional sources, and relevant sources may not have been identified if search terms were not present in the abstract, title, or keywords. Due to the Anglocentric nation inclusion criteria and focus on English language papers, the results of this review are likely most relevant in English-speaking contexts. Finally, the literature search included the years 2020 and 2021, in which the COVID-19 pandemic occurred, meaning that more current sources may have included the impact of the pandemic in their results.

## 5. Conclusions

Of the 97 studies found in this scoping review, most of them represented North American contexts, and focused on police officers, followed by correctional officers, paramedics, and career firefighters. With research gathered from the years 2000–2021, the pace of publication was most brisk in the last 5 years. Overall, among all PSP groups, lack of supervisor support was identified as the most common negative organizational factor while co-worker support was identified as the most common positive organizational factor.

Although the aim of our review was to discover organizational factors among PSP groups, personal factors were present at the highest frequency in the literature found, followed by organizational factors. It is also of note that negative factors were more prominent overall than positive factors, which may indicate how studies are being constructed as well as the challenges of public safety work itself. It is clear based on the evidence that mental health challenges among PSP populations are complex and multifaceted as they are a result of the interactions between operational, personal, and organizational factors.

### Future Research

Findings from this review will allow PSP, their unions, and public safety organizations to better understand the impact of organizational factors on PSP mental health. Additionally, this review has revealed gaps in existing research that can inform future studies in this domain. A similar review of non-Anglocentric countries could be completed to allow for comparison of these factors in public safety organizations across cultural contexts. Evaluation of the studies was beyond the scope of this review and is recommended in future reviews to understand the impact of organizational factors as this body of research continues to expand. Additionally, a model of organizational factors could be created and tested against mental health outcomes for public safety organizations and is a planned next step for this group of researchers.

## Figures and Tables

**Table 1:** Study Characteristics and Factors.

#	Study ID	Country	Population	Study Design	Operational Factors (Negative)	Personal Factors (Negative)	Organizational Factors (Negative)	Operational Factors (Positive)	Personal Factors (Positive)	Organizational Factors (Positive)
[17]	Amendola 2011	United States	Police officer	Randomised controlled trial	N/A	N/A	N/A	N/A	N/A	N/A
[18]	Angehrn 2021	Canada	Police officer	Cross sectional study	N/A	Substance misuse; Poor sleep; Gender	N/A	N/A	Social support; Family support	N/A
[19]	Armstrong 2016	Australia	Firefighter (career)	Cross sectional study	Administrative duties; High workload; Exposure to critical incidients (generic)	Lack of social support; Lack of family support	Shift work (other model)	N/A	Job satisfaction/meaning	Recognition of good work
[20]	Barnes-Farrell 2018	United States	Correctional officer	Quasi-experimen-tal design	N/A	Age	Overtime hours	N/A	N/A	N/A
[21]	Beauchamp 2021	United States	Police officer	Case control study	Risk of own injury; Negative public perception of career; Length of service	Lack of family support; Work/life/family conflict; Fatigue; Major business readjustment	Managing with supervisors; Internal investigations	N/A	Family support	N/A
[22]	Bennett 2005	UK	Paramedic; EMT	Cross sectional study	Exposure to critical incidents (generic); Unpredictable nature of work; Incidents involving children; Length of service	Work/life/family conflict; Health conditions (mental); Intrusive memories or thoughts; Dissociation at the time of traumatic event	Lack of coworker support	N/A	Gender	N/A
[23]	Biggs 2014	Australia	Police officer	Longitud-inal design	Exposure to natural disaster	Job dissatisfaction; Experiencing personal property damage or loss	Limited resources to perform the work; Workplace culture	N/A	Job satisfaction/meaning	Supervisor support; Autonomy; Workplace culture; Team dynamics; Involvement in major operations
[24]	Birze 2021	Canada	Communications officer	Case control study	Exposure to critical incidents (generic); Workplace stress	Health conditions (mental); Surface acting	Shift work (other model); Lack of organizational support; Organizational pressure	N/A	N/A	N/A
[25]	Bourbonnais 2005	Canada	Correctional officer	Cross sectional study	Psychological demands; Effort/reward imbalance	Gender	Interpersonal conflict (colleague); Interpersonal conflict (supervisor); Lack of supervisor support; Lack of coworker support; Lack of input in decision-making	N/A	N/A	N/A
[26]	Buden 2016	United States	Other: Correctional supervisors	Cross sectional study	N/A	Lack of coping skills; Health conditions (physical); Health conditions (mental); Poor diet, Burnout	N/A	N/A	Job satisfaction or meaning	Coworker support
[27]	Caputo 2015	United States	Firefighter (career)	Quasi-experimen-tal design	N/A	N/A	N/A	N/A	Work/life/family balance; Job satisfaction or meaning; Adequate sleep; Time off, Reduced burnout	Shift work (48 h model)
[5]	Carleton 2020	Canada	Police officer; RCMP/federal police; Firefighter (career); Firefighter (volunteer); Paramedic; Correctional officer; Communications officer; Dispatcher	Cross sectional study	Administrative duties; Exposure to critical incidents (generic); Risk of own injury; Negative public perception of career; Occupation-related health issues; Interacting with the court system; Working alone at night	Lack of family support; Work/life/family conflict; Fatigue; Inability to “turn off”; Pressure to prove oneself; Job-related stigma impacting friends/family; Staying in good physical condition; Eating healthy at work; Upholding a public image	Policy and procedure changes; Overtime hours; Limited resources to perform the work; Lack of training; Understaffing; Interpersonal conflict (colleague); Interpersonal conflict (supervisor); Stigma and barriers to seeking help; Organizational unfairness or lack of justice; Leadership issues; Workplace culture; Favouritism; Volunteering free time; Internal investigations; Surveillance on the job	N/A	Family support	N/A
[28]	Cash 2019	United States	EMT	Cross sectional study	N/A	Job dissatisfaction	Interpersonal conflict (colleague); Interpersonal conflict (supervisor); Workplace culture; Bullying	N/A	N/A	N/A
[29]	Cavallari 2021	United States	Other: Correctional supervisors	Cross sectional study	High workload; Psychological demands; Unpredictability	Poor sleep; Health conditions (mental); Burnout	Shift work (other model); Lack of time off; Long work hours; Overtime hours; Scheduling challenges	N/A	Social support	N/A
[30]	Chan 2020	Canada	Police officer	Cross sectional study	Administrative duties; Negative public perception of career	Fatigue; Health conditions (mental); Health behaviours	Lack of supervisor support; Organizational unfairness or lack of justice; Leadership issues; Organizational pressure	N/A	N/A	N/A
[31]	Charman 2021	UK	Police officer	Qualitative research	High workload	Work/life/family conflict; Job dissatisfaction; Health conditions (physical); Health conditions (mental)	Shift work (12 h model); Lack of autonomy; Lack of supervisor support; Organizational unfairness or lack of justice; Leadership issues; Workplace culture; Bullying; Lack of recognition for good work	N/A	N/A	N/A
[32]	Clements 2021	UK	Correctional officer	Cross sectional study	High workload; Experiencing violence;	Emotional exhaustion; Burnout	Understaffing; Lack of supervisor support; Stigma and barriers to seeking help; Organizational unfairness or lack of justice	N/A	N/A	N/A
[33]	Collins 2003	UK	Police officer	Cross sectional study	High workload	Lack of coping skills; Work/life/family conflict; Job dissatisfaction; Inability to “turn off”; Type A personality	Lack of autonomy; Lack of supervisor support; Workplace culture	N/A	N/A	N/A
[34]	Courtney 2013	Australia	Paramedic	Cross sectional study	N/A	Poor sleep; Fatigue; Health conditions (mental)	Shift work (10 h model); Shift work (14 h model)	N/A	N/A	N/A
[35]	Craddock 2022	United States	Police officer	Cross sectional study	Exposure to critical incidents (generic); Risk of own injury; Risk of own death; Incidents involving children	Health conditions (physical); Health conditions (mental)	Stigma and barriers to seeking help; Workplace culture	N/A	N/A	N/A
[36]	Crowe 2018	United States	Paramedic; EMT	Cross sectional study	High workload	Gender; Burnout	Private organizations	N/A	N/A	N/A
[37]	Demou 2020	UK	Police officer	Qualitative research	Work overload; Exposure to critical incidents (generic); Witnessing accidental death or murder (coworker); Negative public perception of career	Work/life/family conflict	Role ambiguity; Policy and procedure changes; Overtime hours; Lack of training; Stigma and barriers to seeking help; Lack of recognition for good work; Leadership issues; Workplace culture; Bullying	N/A	Organizational belongingness	Coworker support; Team dynamics; Humour
[38]	Dir 2019	United States	Correctional officer	Cross sectional study	N/A	N/A	Stigma and barriers to seeking help; Workplace culture; Lack of input in decision-making	N/A	N/A	N/A
[39]	Dollard 2012	Australia	Police officer	Longitud-inal design	Exposure to critical incidents (generic)	Lack of coping skills	Stigma and barriers to seeking help; Bullying	N/A	N/A	Coworker support; Supervisor support; Access to mental health specialists; Informal debriefing; Workplace culture
[40]	Donnelly 2016	Canada	Paramedic	Cross sectional study	Exposure to critical incidents (generic); Risk of own injury	Work/life/family conflict; Fatigue; Health conditions (mental); Inability to “turn off”; Lack of social support	Shift work (other model); Lack of breaks (while working)	N/A	N/A	N/A
[41]	Dugan 2021	United States	Correctional officer	Qualitative research	Exposure to critical incidents (generic); Witnessing injuries; Witnessing suicide death; Threats/risk of violence; Physical injury; Experiencing violence; Negative public perception of career	Lack of coping skills; Substance misuse; Work/life/family conflict; Poor sleep; Lack of family support seeking; Poor health literacy	Workplace culture	N/A	Family support	N/A
[42]	Dyal 2021	United States	Firefighter (career)	Cross sectional study	N/A	Poor sleep	N/A	N/A	Adequate sleep	N/A
[43]	Eades 2020	Australia	Other: Immigration detention staff	Qualitative research	Work overload; Witnessing injuries; Witnessing suicide attempt; Secondary trauma; Risk of own injury; Interpersonal conflict (patient/prisoner/public)	Lack of coping skills; Lack of family support; Substance misuse; Poor sleep; Self-stigma; Controlling detainees	Lack of time off; Overtime hours; Lack of training; Lack of autonomy; Interpersonal conflict (supervisor); Lack of supervisor support; Stigma and barriers to seeking help; Lack of recognition for good work; Leadership issues; Surveillance on the job; Confidentiality	N/A	Work/life/family balance; Good physical health; Job satisfaction or meaning	Access to peer support (formal program); Coworker support; Adequate training
[44]	El Sayed 2019	United States	RCMP/federal police	Qualitative research	Administrative duties; Work overload; Risk of own injury; Risk of own death; Threats/risk of violence; Negative public perception of career; Boredom; Unpredictability	Lack of family support	Policy and procedure changes; Lack of supervisor support; Lack of recognition for good work; Conflicting information	N/A	N/A	N/A
[45]	Ellison 2020	United States	Correctional officer	Cross sectional study	High workload; Witnessing injuries; Threats/risk of violence	N/A	Role conflict; Understaffing; Inmate to officer ratio	N/A	Family support	Coworker support; Supervisor support; Autonomy
[46]	Fortune 2018	United States, Europe, Canada	Police investigator (online)	Qualitative research	High workload; Exposure to upsetting online content	Spousal/marital challenges; Self-stigma; Intrusive memories or thoughts; Suicidal thoughts/risk; Hypersensitivity around children; Negative affect	Limited resources to perform the work; Lack of training	N/A	Coping skills; Family support; Work/life/family balance; Healthy behaviours	Coworker support; Adequate training; Varied workload
[47]	Galbraith 2021	UK	Police officer; Dispatcher	Cross sectional study	High workload	Substance misuse; Work/life/family conflict; Health conditions (physical); Health conditions (mental); Gender	Role conflict; Lack of autonomy; Interpersonal conflict (colleague); Lack of supervisor support; Lack of coworker support; Managerial change	N/A	N/A	Supervisor support
[48]	Genest 2021	Canada	Correctional officer	Cross sectional study	N/A	Work/life/family conflict; Spousal/marital challenges; Pre-work trauma	Scheduling challenges; Lack of access to mental health supports; Denied access to mental health supports; Stigma and barriers to seeking help; Bullying	N/A	Family support; Pets; Emotional collateral damage; Medication	Access to mental health specialists; On leave
[49]	Geoffrion 2017	Canada	Police officer	Cross sectional study	Experiencing violence	Gender	Stigma and barriers to seeking help; Workplace culture	N/A	N/A	Coworker support; Supervisor support; Normalizing workplace violence
[50]	Hartley 2014	United States	Police officer	Cross sectional study	Administrative duties; Risk of own injury; Threats/risk of violence; Workplace stress	Lack of coping skills; Job dissatisfaction; Poor sleep; Health conditions (mental)	Lack of supervisor support; Lack of coworker support	N/A	N/A	N/A
[51]	Houdmont 2016	UK	Police officer	Cross sectional study	Exposure to critical incidents (generic); Experiencing violence	Health conditions (mental); Emotional exhaustion; Gender; Depersonalization; Years of experience	Long work hours; Overtime hours; Lack of training; Stigma and barriers to seeking help	N/A	N/A	N/A
[52]	Huddle-ston 2006	New Zealand	Police officer	Longitud-inal design	Exposure to critical incidents (generic)	Health conditions (physical); Health conditions (mental); Pre-work trauma	Leadership issues; Poor communication	N/A	N/A	Recognition of good work; Empowerment; Having responsibility
[53]	Huddle-ston 2007	New Zealand	Police officer	Cohort study	Administrative duties; Work overload; Exposure to critical incidents (generic); Fast-paced environment	Health conditions (mental)	Shift work (other model); Limited resources to perform the work	N/A	N/A	N/A
[54]	Hurtado 2018	United States	Other: Parole/probation officer (PPO)	Cross sectional study	N/A	N/A	Scheduling challenges; Lack of autonomy	N/A	N/A	N/A
[55]	Jahnke 2019	United States	Firefighter (career)	Cross sectional study	N/A	Job dissatisfaction; Health conditions (mental); Gender; Sexual orientation; Race	Workplace culture; Bullying	N/A	N/A	N/A
[56]	Jones 2018	United States	Firefighter (career); Firefighter (volunteer); Paramedic; EMT	Cross sectional study	Rank; Department setting	Gender; Relationship status	Shift work (48 h model)	Department setting; Rank	Relationship status	Shift work (12 h model); Shift work (14 h model)
[57]	Juniper 2010	UK	Police officer	Cohort study	Boredom; Rank	Work/life/family conflict; Poor diet	Overtime hours; Lack of recognition for good work; Leadership issues; Policy and procedure changes; Inadequate Facilities	N/A	N/A	N/A
[58]	Kimbrel 2011	United States	Firefighter (career)	Measuring psychometric properties	N/A	N/A	N/A	N/A	N/A	N/A
[59]	Kinman 2016	UK	Correctional officer	Cross sectional study	High workload	Job dissatisfaction; Health conditions (mental)	Role ambiguity; Policy and procedure changes; Lack of autonomy; Lack of supervisor support; Lack of coworker support; Bullying; Ineffective management of change	N/A	N/A	N/A
[60]	Kyprian-ides 2021	UK	Police officer	Cross sectional study	N/A	Self-legitimacy	N/A	N/A	Organizational belongingness	Organizational fairness or justice; Workplace culture; Staff aligning with organizations goals/value/mission
[61]	Kyron 2022	Australia	Police officer; Firefighter (career); Paramedic; EMT; Other: State emergency service employees	Cross sectional study	Length of service	Self-stigma	N/A	N/A	N/A	N/A
[62]	Kyron 2021	Australia	Police officer; Firefighter (career); Paramedic; Other: State emergency service employees	Cross sectional study	N/A	Substance misuse	Worker’s compensation system challenges	N/A	Youth	Supervisor support; Positive perception of mental health supports; Workplace culture
[63]	Lambert 2010	United States	Correctional officer	Cross sectional study	Rank	Work/life/family conflict; Perceived dangerousness	Role conflict; Role ambiguity	N/A	N/A	N/A
[64]	Langtry 2021	UK, Ireland	Firefighter (career)	Cross sectional study	Exposure to critical incidents (generic); Risk of own death; Rank	Pre-work trauma	N/A	N/A	N/A	N/A
[65]	Lavigne 2010	Canada	Correctional officer	Cross sectional study	Effort/reward imbalance	Substance misuse	Interpersonal conflict (colleague); Lack of coworker support; Bullying	N/A	N/A	N/A
[66]	Lawson 2022	United States	Police officer	Cross sectional study	High workload; Threats/risk of violence; Experiencing violence	Noble-cause corruption beliefs	Interpersonal conflict (colleague); Lack of coworker support; Bullying	N/A	N/A	Good leadership
[67]	Lerman 2022	United States	Correctional officer	Cross sectional study	Exposure to critical incidents (generic); Experiencing violence	Lack of coping skills; Poor sleep; Health conditions (mental); Burnout, Suicide thoughts/risk	Long work hours; Overtime hours; Lack of training; Stigma and barriers to seeking help	N/A	N/A	Supervisor support
[68]	Lucas 2012	United States	Police officer	Cross sectional study	Experiencing violence; Killing in the line of duty	N/A	Lack of training	N/A	N/A	N/A
[69]	Ma 2015	United States	Police officer	Cross sectional study	Effort/reward imbalance; Risk of own injury; Threats/risk of violence; Negative public perception of career; Workplace stress	Work/life/family conflict; Stress	Overtime hours; Lack of autonomy; Lack of input in decision-making	N/A	N/A	N/A
[70]	Ma 2019	United States	Police officer	Cross sectional study	Effort/reward imbalance; High workload; Risk of own injury; Threats/risk of violence; Negative public perception of career; Workplace stress	Work/life/family conflict; Poor sleep; Psychological stressors	Lack of supervisor support; Lack of input in decision-making	N/A	N/A	N/A
[71]	Maguen 2009	United States	Police officer	Cohort study	Exposure to critical incidents (generic)	Race; Negative life events	Limited resources to perform the work; Lack of supervisor support; Lack of coworker support; Workplace culture	N/A	Gender; Race	N/A
[72]	Mahfood 2013	United States	Correctional officer	Cross sectional study	Work overload	No children	Role conflict; Role ambiguity; Prison physical condition	Perceived threat to safety	N/A	N/A
[73]	Mahony 2001	Australia& UK	Paramedic	Qualitative research	High workload; Work overload; Negative public perception of career	Job dissatisfaction	Lack of breaks (while working); Long work hours; Limited resources to perform the work; Lack of autonomy; Lack of supervisor support; Lack of recognition for good work; Organizational unfairness or lack of justice; Leadership issues; Lack of input in decision-making; Resource wasting, Lack of de-compression time, Finances; Absenteeism	N/A	N/A	N/A
[74]	Mahony 2005	UK	Paramedic	Qualitative research	Administrative duties; High workload	N/A	Shift work (12 h model); Lack of breaks (while working); Understaffing; Lack of autonomy; Workplace culture; Lack of input in decision-making; Surveillance on the job	N/A	N/A	N/A
[75]	Miller 2018	United States	Police officer; Police investigator (online); Firefighter (career); Firefighter (volunteer); Dispatcher; Other: EMS	Cross sectional study	N/A	Education	Lack of coworker support; Lack of access to mental health supports; Employment status, Cross-training	N/A	Resilience; Education	Coworker support; Organizational support; Adequate training; Cross-training (working in other fields); Informal debriefing
[76]	Murphy 2002	United States	Firefighter (career)	Longitud-inal design	Threats/risk of violence; Boredom	Substance misuse	Limited resources to perform the work; Interpersonal conflict (colleague); Interpersonal conflict (supervisor); Lack of recognition for good work	N/A	Good physical health; Lack of smoking	N/A
[77]	Navarro Moya 2020	Canada	EMT	Qualitative research	Work overload; Risk of own injury; Risk of own death; Threats/risk of violence; Workplace stress; Incidents involving children	Work/life/family conflict; Health conditions (physical); Health conditions (mental); Emotional exhaustion; Disalignment of job expectations and reality	Shift work (14 h model); Shift work (other model); Lack of breaks (while working); Limited resources to perform the work; Lack of training; Understaffing; Lack of supervisor support; Stigma and barriers to seeking help; Leadership issues; Fear of losing job; Surveillance on the job; Interpersonal conflict (colleague)	N/A	Family support; Job satisfaction or meaning	Coworker support
[78]	Neylan 2002	United States	Police officer	Quasi-experimen-tal design	Exposure to critical incidents (generic); Workplace stress	N/A	Shift work (other model)	N/A	N/A	N/A
[79]	Noor 2019	United States	Firefighter (career)	Cross sectional study	Length of service	Gender; Relationship status	Lack of access to mental health supports	N/A	Gender; Relationship status	N/A
[80]	Norman 2022	Canada	Correctional officer	Qualitative research	Administrative duties; High workload; Secondary trauma; Interpersonal conflict (patient/prisoner/public)	Job dissatisfaction; Health conditions (mental); Pre-work trauma; Burnout	Limited resources to perform the work; Lack of training; Understaffing; Interpersonal conflict (colleague); Interpersonal conflict (supervisor); Lack of supervisor support; Workplace culture; Team dynamics	N/A	N/A	N/A
[81]	Nurse 2003	UK	Correctional officer	Qualitative research	Threats/risk of violence	Job dissatisfaction; Self-stigma	Understaffing; Lack of supervisor support; Leadership issues; Workplace culture; Inmate population, Poor communication; Lack of care for prisoners	N/A	N/A	N/A
[82]	Padilla 2020	United States	Police officer	Cross sectional study	Administrative duties; Exposure to critical incidents (generic); Risk of own injury; Experiencing violence; Negative public perception of career; Rapid critical decisions; Workplace stress; Court appearances; Interpersonal conflict (patient/prsioner/public); Rank	Work/life/family conflict; Physical fitness	Role ambiguity; Shift work (other model); Policy and procedure changes; Overtime hours; Limited resources to perform the work; Understaffing; Lack of autonomy; Interpersonal conflict (colleague); Lack of supervisor support; Lack of recognition for good work; Leadership issues; Discrimination; Judicial/correctional system ineffectiveness; Role conflict, Workplace culture; Lack of recognition for good work; Unfamiliar duties; Competition for advancement	Tenure	Race	N/A
[83]	Payne 2019	UK	Firefighter (career)	Cross sectional study	N/A	N/A	Interpersonal conflict (colleague); Interpersonal conflict (supervisor); Limited resources to perform the work	N/A	Coping skills; Ability to detach; Lack of rumination	Autonomy; Role clarity
[84]	Peterson 2019	Canada, United States	Police officer	Cross sectional study	N/A	Poor sleep; Fatigue	Shift work (other model); Overtime hours	N/A	N/A	Autonomy
[85]	Pisarski 2002	Australia	EMT	Cross sectional study	High workload	Lack of coping skills; Health conditions (physical); Health conditions (mental)	Shift work (other model)	N/A	Coping skills; Good physical health; Good mental health	Coworker support; Supervisor support
[86]	Pyper 2016	Australia	EMT	Mixed methods	Administrative duties; Witnessing accidental death or murder (civilian); Incidents involving children; Working with critically ill patients; Community expectations; Long travel distances	Poor sleep; Fatigue	Interpersonal conflict (colleague); Leadership issues; Lack of coworker support	N/A	N/A	N/A
[87]	Raper 2020	Australia	Police officer	Cohort study	N/A	N/A	N/A	N/A	N/A	Organizational resources of strategic alignment
[88]	Reuter 2017	United States	Paramedic	Mixed methods	Psychological demands	Health behaviours	Policy and procedure changes; Limited resources to perform the work; Lack of supervisor support; Lack of coworker support; Poor communication	N/A	N/A	N/A
[89]	Ricciardelli 2020a	Canada	Correctional officer	Qualitative research	Exposure to critical incidents (generic)	Job dissatisfaction; Self-stigma	Lack of access to mental health supports; Stigma and barriers to seeking help; Lack of recognition for good work; Fixed term employment	N/A	N/A	N/A
[90]	Ricciardelli 2020b	Canada	Police officer; RCMP/federal police; Firefighter (career); Firefighter (volunteer); Paramedic; EMT; Correctional officer; Dispatcher; Other: Canadian boarder services, Coast guard, Transit police	Mixed methods	Exposure to critical incidents (generic)	Health conditions (mental); Misaligment of job expectations and reality	Lack of training; Understaffing; Lack of coworker support; Stigma and barriers to seeking help	N/A	N/A	N/A
[91]	Ricciardelli 2020c	Canada	Firefighter (career); Paramedic; Communications officer; Other: Federal police, Municipal/provincial police	Qualitative research	Administrative duties; Negative public perception of career; Departmental setting	Inability to “turn off”	Policy and procedure changes; Limited resources to perform the work; Understaffing; Interpersonal conflict (colleague); Interpersonal conflict (supervisor); Lack of supervisor support; Workplace culture; Bullying	N/A	N/A	N/A
[92]	Ricciardelli 2020d	Canada	Correctional officer	Qualitative research	Exposure to critical incidents (generic); Witnessing accidental death or murder (civilian); Witnessing injuries; Witnessing suicide death; Risk of own injury; Threats/risk of violence; Physical injury; Experiencing violence; Exposure to bodily fluid	Lack of coping skills; Health conditions (mental)	Policy and procedure changes; Lack of supervisor support; Lack of coworker support; Workplace culture; Bullying; Poor communication	N/A	Desensitization	N/A
[93]	Ricciardelli 2021	Canada	Correctional officer	Cross sectional study	Intolerance for uncertainty; Rank	N/A	N/A	Role	N/A	N/A
[94]	Setlack 2020	Canada	Firefighter (career); Paramedic	Cohort study	Risk of own death; Threats/risk of violence; Physical injury	Post-traumatic cognition	N/A	N/A	N/A	N/A
[95]	Smith 2018	United States	Firefighter (career)	Cross sectional study	Workplace stress	Work/life/family conflict	Limited resources to perform the work; Poor communication	N/A	N/A	N/A
[96]	Smith 2019	United States	Firefighter (career); Paramedic	Cross sectional study	High workload; Work overload; Workplace stress	Work/life/family conflict	Limited resources to perform the work; Understaffing; Lack of autonomy; Lack of input in decision-making	N/A	N/A	Role clarity
[97]	Smith 2020	United States	Firefighter (career)	Cross sectional study	N/A	Burnout	N/A	N/A	N/A	Safe work practices; Safety citizenship behavior
[98]	Sofiano-poulos 2011	Australia	Paramedic	Cross sectional study	High workload	Poor sleep; Fatigue; Health conditions (mental); Daytime sleepiness	Shift work (other model); Long work hours	N/A	N/A	N/A
[99]	Soh 2016	UK	EMT	Cross sectional study	N/A	N/A	N/A	N/A	N/A	N/A
[100]	Steel 2021	UK	Police officer	Cross sectional study	Rank; Workplace stress	Health conditions (mental); Pre-work trauma	Lack of supervisor support	Role	Job satisfaction or meaning; Adequate sleep	N/A
[101]	Steinkopf 2018	United States	Dispatcher	Cross sectional study	Workplace stress	Lack of resilience; Health conditions (mental); Negative affect	Lack of supervisor support; Team dynamics	N/A	Social support; Personal strengths; Spiritual change; New possibilities	N/A
[102]	Tehrani 2018	UK	Other: Child abuse investigator (CAI)	Cross sectional study	N/A	Pre-work trauma; Gender; Tenure	N/A	N/A	Workability	N/A
[103]	Trounson 2016	Australia	Correctional officer	Cross sectional study	Exposure to critical incidents (generic); Threats/risk of violence; Workplace unpredictability	Inability to “turn off”	Lack of breaks (while working); Absenteeism	N/A	N/A	N/A
[104]	Trounson 2019	Australia	Correctional officer	Cross sectional study	Exposure to critical incidents (generic); Threats/risk of violence; Workplace unpredictability	Job dissatisfaction; Inability to “turn off”; Poor psychological wellbeing	Lack of breaks (while working); Absenteeism	N/A	N/A	N/A
[105]	Trounson 2021	Australia	Correctional officer	Qualitative research	Working with incarcerated family members	Substance misuse	Role conflict; Lack of access to mental health supports; Workplace culture	N/A	Work/life/family balance; Job satisfaction or meaning; Subjective wellbeing	Cultural engagement
[106]	Tsai 2018	United States	Police officer	Cross sectional study	N/A	Gender; Education; Tenure; Rank	Workplace culture; Lack of access to mental health supports	N/A	Gender; Race; Rank	N/A
[107]	Tuckey 2010	Australia	Police officer	Cross-sectional longitudinal	N/A	Health conditions (physical); Health conditions (mental)	Bullying	N/A	N/A	N/A
[108]	Violanti 2014	United States	Police officer	Cross sectional study	Administrative duties; Witnessing accidental death or murder (coworker); Negative public perception of career; Workplace stress	N/A	Understaffing; Interpersonal conflict (colleague); Interpersonal conflict (supervisor)	N/A	Resilience	N/A
[109]	Violanti 2016	United States	Police officer	Cross sectional study	N/A	Hopelessness	Administrative practices	N/A	N/A	N/A
[110]	Violanti 2018	United States	Police officer	Cross sectional study	High workload; Physical demands	Lack of coping skills; Fatigue	Overtime hours; Lack of recognition for good work; Fear of losing job	N/A	Resilience	N/A
[111]	Walters 2022	United States	Correctional officer	Cross sectional study	Interpersonal conflict (patient/prisoner/public)	Work/life/family conflict; Gender; Age	Lack of supervisor support; Lack of coworker support; Finances	N/A	N/A	N/A
[112]	Werner-de-Sondberg 2021	UK	Police officer; Correctional officer	Mixed methods	Public/private system	Negative affect	Workplace culture; Shift work (12 h model)	N/A	Intolerance for ambiguity; Energy	Shift work (other model); Workplace culture; Shared leadership

**Table 2:** Factor Frequencies.

Factors	Negative	Positive	Total
Operational	119	7	126
Personal	206	67	273
Organizational	145	63	208
Total	470	137	607

## Appendix A

**Table A1:** Sample Search Strategy.

#	Searches	Results
1	exp Organizational Culture/	18,415
2	exp organizational policy/	14,452
3	exp Organizational Innovation/	27,414
4	exp Efficiency, Organizational/	22,250
5	((“work environment” or workplace or “work place” or “work force” or organizational or Organisational or institutional or union or work*) adj2 (trust* or honesty or fairness or culture or value* or belief or believes* compassion* or satisfaction* or burnout* or stress* or attitud* or promotion* or understanding or polic* or leadership* or factor* or consequence* or bullying or theor* or behaviour* or behavior* or framework* or development* or communication* or engagement* or micromanagement or workload* or fatigue or resilience or control* or autonom* or conflict)).ab,kw,ti.	107,522
6	1 or 2 or 3 or 4 or 5	177,814
7	exp emergency responders/or exp emergency medical technicians/or exp firefighters/or exp police/	13,896
8	exp Military Personnel/	42,118
9	exp Correctional Facilities/	10,607
10	exp Emergency Medical Service Communication Systems/	1833
11	(Public service personnel* or Public safety personnel* or public service agenc* or police or correctional or traffic* or paramedic* or securit* or dispatcher* or firefighter* or RCMP or Public safety communication* or military or ambulance or first responder* or Officer* or enforcement).ab,kw,ti.	280,351
12	7 or 8 or 9 or 10 or 11	316,901
13	Mental Health/	48,663
14	exp Psychological Trauma/or exp Stress, Psychological/or exp Psychological Distress/	148,901
15	exp Burnout, Professional/	14,363
16	“Quality of Life”/	226,476
17	(Mental health or wellness or well-being or stress* or mood or panic* or anxiet* or depression or psychological or trauma or mental hygiene).ab,kw,ti.	1,717,364
18	13 or 14 or 15 or 16 or 17	1,922,243
19	6 and 12 and 18	1929

Database: Ovid MEDLINE(R), Ovid MEDLINE(R) Daily and Epub Ahead of Print, In-Process & Other Non-Indexed Citations 1946 to November 2021.
